# Performance of deep learning-based algorithm for detection of ileocolic intussusception on abdominal radiographs of young children

**DOI:** 10.1038/s41598-019-55536-6

**Published:** 2019-12-19

**Authors:** Sungwon Kim, Haesung Yoon, Mi-Jung Lee, Myung-Joon Kim, Kyunghwa Han, Ja Kyung Yoon, Hyung Cheol Kim, Jaeseung Shin, Hyun Joo Shin

**Affiliations:** Department of Radiology, Severance Hospital, Research Institute of Radiological Science, Center for Clinical Imaging Data Science, Yonsei University College of Medicine, 50-1 Yonsei-Ro, Seodaemun-Gu, Seoul, 03722 Korea

**Keywords:** Gastroenteritis, Paediatric research

## Abstract

The purpose of this study was to develop and test the performance of a deep learning-based algorithm to detect ileocolic intussusception using abdominal radiographs of young children. For the training set, children (≤5 years old) who underwent abdominal radiograph and ultrasonography (US) for suspicion of intussusception from March 2005 to December 2017 were retrospectively included and divided into control and intussusception groups according to the US results. A YOLOv3-based algorithm was developed to recognize the rectangular area of the right abdomen and to diagnose intussusception. For the validation set, children (≤5 years old) who underwent both radiograph and US from January to August 2018 with the suspicion of intussusception were included. Diagnostic performances of an algorithm and radiologists were compared. Total 681 children including 242 children in intussusception group were included in the training set and 75 children including 25 children in intussusception group were included in the validation set. The sensitivity of the algorithm was higher compared with that of the radiologists (0.76 vs. 0.46, p = 0.013), while specificity was not different between the algorithm and the radiologists (0.96 vs. 0.92, p = 0.32). Deep learning-based algorithm can aid screening of intussusception using abdominal radiography in young children.

## Introduction

Ileocolic intussusception is a common cause of acute abdominal pain in young children. Early diagnosis and reduction are important because delayed treatment can lead to bowel ischemia, perforation, and eventually peritonitis. For diagnosis of intussusception, emergency ultrasonography (US) is warranted in the presence of cyclic irritability, vomiting, or hematochezia^1^. However, because these symptoms are nonspecific and can be present in less than 50% of children with intussusception, frequent emergency US procedures are required for screening of intussusception^2^.

Abdominal radiograph is usually performed before US examination in children with abdominal symptoms. A crescent or target sign is a classic imaging finding for intussusception on radiography^3^. However, the sensitivity of radiography for diagnosis of intussusception is quite low (45%); because of this, it is usually not recommended as the sole basis for diagnosis of intussusception itself^1^. Instead, radiography is usually performed in children to exclude other diagnoses or to identify complications such as peritonitis. In contrast, US offers high sensitivity (97.9%) and specificity (97.8%) for detection of intussusception, and frequent US examinations are performed for screening of intussusception in emergency settings^2^. However, in one recent study, 86% of children who underwent US for suspicion of intussusception had negative results^2^. Therefore, if there is a more effective method for screening children for intussusception using radiograph, emergency US could be performed more selectively.

Deep learning represents an increasingly disruptive technology and is being adopted earlier in radiology for reasons such as detection, segmentation, and classification using imaging data^4–7^. Also, among children, deep learning-based algorithms have been used for analysis of bone and chest radiographs for assessment of bone age, fracture, effusion, and differentiation of various projection views^4,8–10^. However, there is no study that has assessed whether deep learning could aid with detection of focal lesions on abdominal radiographs of young children. If an algorithm could aid in detection of ileocolic intussusception using radiography, it could be used to supplement the screening of children for emergency US or for referral to a tertiary hospital for reduction or operation for intussusception.

Therefore, the purpose of this study was to develop and test the performance of a deep learning-based algorithm to detect ileocolic intussusception using abdominal radiography in young children.

## Results

**Figure 1:**
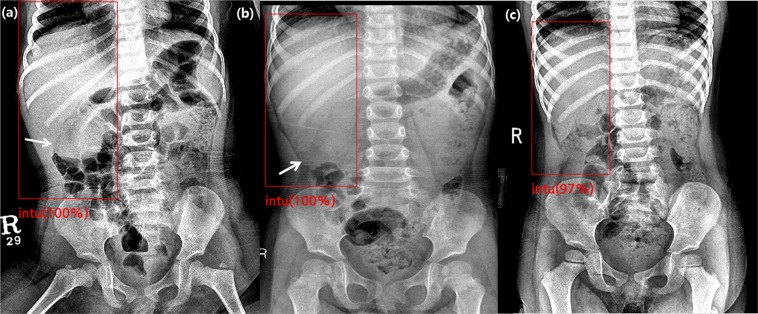
True-positive cases found by the algorithm in the validation set. **(a)** A supine abdominal radiograph of a 26-month-old female with abdominal pain. Her initial radiograph shows a round opacity in the right upper abdomen (arrow) and the ileocolic intussusception was confirmed on US. To train the algorithm, an ROI using the upper boundary of the right diaphragm, lower boundary of the iliac crest, medial boundary of the spine, and lateral boundary of the lateral rib cage was drawn. This image was actually included in the validation set. The algorithm and two out of four radiologists correctly diagnosed intussusception, while the other two radiologists suggested it negative for intussusception. **(b)** An abdominal radiograph of a 28-month-old female with intussusception. One radiologist labeled it positive for intussusception based on the round opacity in the right upper abdomen (arrow) and the algorithm reported a true-positive result. **(c)** An abdominal radiograph of a 30-month-old girl with intussusception. The algorithm reported a true-positive result, while all four radiologists reported false-negative results.

**Figure 2:**
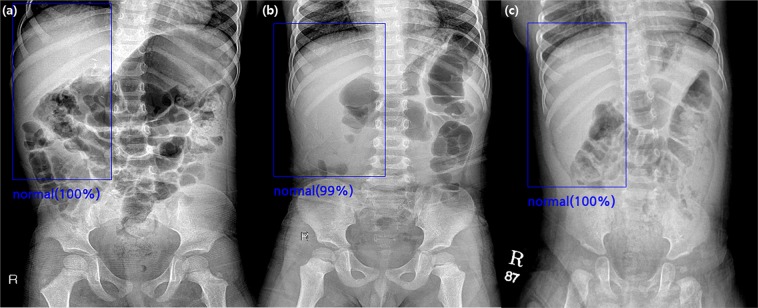
True-negative cases found by the algorithm in the validation set. **(a)** A supine abdominal radiograph of a 36-month-old girl without intussusception. All four radiologists and the algorithm reported true-negative results because there was no round opacity obscuring bowel gas in the right abdomen. **(b)** A supine abdominal radiograph of a 48-month-old boy without intussusception. Two radiologists reported false-positive results, while the algorithm and the other two radiologists reported true-negative results. **(c)** A supine abdominal radiograph of an 11-month-old boy without intussusception. Four radiologists and the algorithm all reported true-negative results.

**Figure 3:**
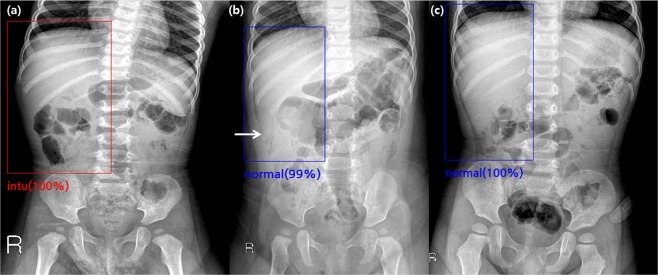
False-positive and false-negative cases found by the algorithm in the validation set. **(a)** A supine abdominal radiograph of a 15-month-old boy without intussusception. One radiologist and the algorithm reported false-positive results, while the other three radiologists reported true-negative results. **(b)** A supine abdominal radiograph of a 10-month-old boy with intussusception. One radiologist and the algorithm reported false-negative results, while the other three radiologists reported true-positive results. **(c)** A supine abdominal radiograph of a 29-month-old boy with intussusception. All of the four radiologists and the algorithm reported false-negative results.

A total of 681 children was included in the training set, with 242 children (M:F = 151:91, mean age: 21.2 ± 13.4 months) in the intussusception group and 439 children (M:F = 256:183, mean age: 21.4 ± 14.4 months) in the control group. In the temporary independent validation set, a total of 75 children was included with 25 children (M:F = 12:13, mean age: 21.4 ± 11.5 months) in the intussusception group and 50 children (M:F = 33:17, mean age: 24.2 ± 17.5 months) in the control group. The algorithm identified intussusception correctly in 19 of 25 true-positive cases (Fig. 1) and suggested negative findings correctly in 48 of 50 children without intussusception (Fig. 2). Among the six cases of false-negative results produced by the algorithm, three cases also were labeled with false-negative results from the four radiologists. All of the six cases had intussusception in the right side of the abdomen, around the subhepatic space on US. For the two cases of false-positive results produced by the algorithm, one radiologist also suggested a false-positive result for each case, respectively (Fig. 3).

The sensitivities and specificities of the algorithm and radiologists are presented and compared in Table 1. The sensitivity of the algorithm using the validation set was 0.76 with a 95% confidence interval (CI) of 0.56 to 0.89, while the specificity was 0.96 with a 95% CI of 0.85 to 0.99. When we performed 5-fold cross validation using the training set, the sensitivities of the five subsets were subsequently 0.750, 0.750, 0.708, 0.729, and 0.833. In addition, the specificities of the five subsets were subsequently 0.943, 0.943, 0.920, 0.931, and 0.943. The average sensitivity from cross validation was 0.754 and did not differ from the sensitivity of the algorithm using the validation set (p = 0.925). The average specificity from cross validation was 0.936 and again, did not differ from that of the algorithm using the validation set (p = 0.492).

**Table 1:** Comparison of diagnostic performances between the algorithm and radiologists for the detection of ileocolic intussusception in abdominal radiographs.

Tests		Sensitivity	Specificity
Algorithm		0.76 (95% CI: 0.56–0.89) [19/25]	0.96 (95% CI: 0.85–0.99) [48/50]
Residents	Reader-averaged	0.36 (95% CI: 0.23–0.51)	0.91 (95% CI: 0.84–0.95)
	1	0.44 [11/25]	0.98 [49/50]
	2	0.28 [7/25]	0.84 [42/50]
p-value between algorithm and residents		0.002	0.264
Faculties	Reader-averaged	0.56 (95% CI: 0.42–0.69)	0.92 (95% CI: 0.84–0.96)
	3	0.68 [17/25]	0.92 [46/50]
	4	0.44 [11/25]	0.92 [46/50]
p-value between algorithm and faculties		0.013	0.336
Overall p-value		0.013	0. 32

Abbreviations: CI, confidence interval.

Values in [] represent true cases among all cases.

Using the validation set, the sensitivity of the four radiologists was 0.46 (95% CI: 0.33–0.60). More specifically, the sensitivity of the two residents was 0.36 (95% CI: 0.23–0.51) and that of the two faculty members was 0.56 (95% CI: 0.42–0.69). Overall, the sensitivity of the algorithm was significantly higher than the sensitivity of the radiologists (p = 0.013). The specificity of the radiologists was 0.92 (95% CI: 0.94–0.96) while the specificity of the residents was 0.91 (95% CI: 0.84–0.95), and that of the faculty members was 0.92 (95% CI: 0.84–0.96). The specificity was therefore not significantly different between the algorithm and radiologists (p = 0.32).

## Discussion

Using pediatric radiographs, deep learning–based algorithms were first developed and applied in bone age assessment^11–14^. Several studies also assessed the performance of algorithms applied to radiographs of children for detection of fracture or joint effusion^10^. A recent investigation demonstrated that deep learning could effectively classify the projection view of chest radiographs including those of children^9^. However, there is no study that has assessed the utility of deep learning for detection of disease in pediatric abdominal radiographs or for certain specific diseases that are important for children in the emergency department.

Intussusception is an important emergency disease to locate in affected young children. This is because delayed diagnosis could lead to bowel ischemia, perforation, and fatalities^15^. Because of the low diagnostic performance of abdominal radiography and nonspecific symptoms in young children who could not express their symptoms properly, abdominal US is an excellent examination modality for the diagnosis of this condition^1,16^. However, one problem is that too many negative US examinations are performed for this reason^2^. In addition, when the hospital does not have US equipment or experienced radiologists or technologists able to perform US on children, referral cases or delayed diagnosis can occur^15^. In a previous study, abdominal radiography could be used as an effective baseline node for identifying children at risk of intussusception with a sensitivity and specificity of 77 and 79%, respectively^17^. Therefore, if deep learning could aid the detection of ileocolic intussusception with abdominal radiographs, it could help screen children who need US or referral to other hospitals without being influenced by the level of experience in pediatric radiographs. This could be a method for reducing unnecessary emergent US examinations and lowering the burden on radiologists and also patients.

Our study revealed that an algorithm trained with 681 abdominal radiographs had higher sensitivity and comparable specificity with those of radiologists when tested using 75 radiographs in different periods. The increased sensitivity here as compared with the radiologists (76% vs. 46%) suggests a potential benefit for utilizing this algorithm in an emergency setting. The algorithm might be used as screening tool for selecting children who need additional US examinations or who need to be referred to tertiary hospitals and this can be done with low false-negative rates. Increased sensitivity is possible because the algorithm can identify the bowel gas pattern of the right side abdomen regardless of other opacities in the remaining region. The target or crescent sign or soft tissue opacity in the right side abdomen is a well-known indication of intussusception. When making a differential diagnosis in infants without intussusception, bowel gas can be scanty or gassy for enteritis, which is the most common cause of abdominal pain in imaged young children, without localized round opacity obscuring bowel gas in the right side abdomen. The algorithm may detect lesions more efficiently without being affected by superimposed bowel gas or internal organ opacities.

Interestingly, three cases where the algorithm had false-negative results also showed false-negative results from all four radiologists. This could be due to the lower sensitivity of the abdominal radiograph itself for diagnosis of intussusception. However, still, one out of four cases could have false-negative results based on this algorithm having a 76% sensitivity. This could also be from the limitation of the abdominal radiograph itself and suggests that US is still needed for the definite diagnosis of the intussusception. However, this algorithm could be used as a supporting tool for clinicians who could not be aided by radiologists or pediatric radiologists, even though this could not be used for definite diagnosis of intussusception.

Utilization of deep learning in pediatric radiology has several limitations. First is the relatively small number of data for consideration in comparison with available adult data^4^. In addition, the images can be different according to age and growth of the children^4^. However, because large datasets are fundamental for development of algorithms^18^, we tried to have as many intussusception radiographs available as possible. Still, for the control group, we could not include all of the negative intussusception cases because of the much larger number of cases included in this group. If we added all of the negative radiographs for the training set, then the algorithm would not be able to predict positive cases appropriately. Therefore, we collected control group members numbering twice that of the intussusception group going backward from December 2017. In addition, we restricted the age of 5 years or younger according to the disease characteristics, because intussusception in children older than this age range is rare. This could increase the diagnostic performance of the algorithm by limiting age ranges and we could utilize smaller datasets. Larger age ranges of children need more data for training the algorithm because children have varying imaging features according to their growth. Moreover, we trained the algorithm to focus on a specific area in the abdomen using an ROI approach in the training set to overcome the limitation of having a small number of images from the children. Even though the algorithm we used did not present a heat map and we could not guess the reason of diagnosis, we focused on the right side of the abdomen where most of ileocolic intussusception cases would occur in and avoid focusing on unnecessary opacities in other parts of the abdomen, lower thorax, and buttocks on radiographs.

There are several limitations in the present study. First, the number of validation sets was small, and the validations were performed in one institution. Therefore, we additionally performed 5-fold cross validation on the training set for internal validation to verify the generalizability and robustness of the algorithm. The performance of the model after 5-fold cross validation using the training set was not significantly different compared to the performance obtained with the validation set. These results support the robustness and generalization of this model. In addition, we performed temporal separation using newly recruited unused data, instead of manual separation or random split according to the TRIPOD statement and previous radiologic research^19,20^. Because this is a preliminary study that is the first to develop an algorithm for intussusception and assess its performance, further external validation with large datasets is needed in the future. Second, for the assessment of diagnostic performance of radiologists, images were given in JPG files for the blind review. Therefore, adjusting the brightness of images as seen in PACS was impossible and could lower the detection rate of intussusception for radiologists. Third, we designed the algorithm to identify the right abdominal area, defined as an ROI in the training set, and to locate intussusception only in this area. If intussusception occurred in an area outside of the ROI, this could lead to a false-negative result. However, in our validation set, all of the six false-negative cases identified using the algorithm had intussusception in the subhepatic space, which was included in the ROI.

In conclusion, this study showed that a novel deep learning–based algorithm could aid the screening of ileocolic intussusception using abdominal radiographs in young children. Further studies with a large number of data from multiple institutions for external validation are needed for utilizing this algorithm for the screening of ileocolic intussusception using radiographs.

## Methods

This retrospective study was approved by the Institutional Review Board of Severance Hospital (Protocol no. 1-2018-0047) and informed consent was waved.

### Image preparation for training

**Figure 4:**
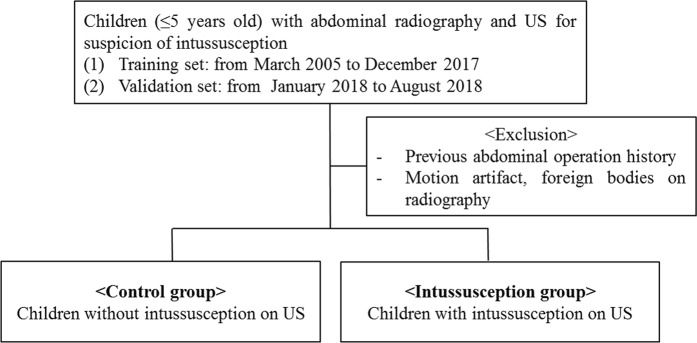
Flow chart for the selection process of the training and validation sets.

Children (≤5 years old) who underwent abdominal US for suspicion of intussusception after supine abdominal radiography according to clinical demands from March 2005 to December 2017 were retrospectively included. Children with previous abdominal operation history, motion artifacts, or foreign bodies (e.g., tubes, catheters, contrasts, button, or accessories) on abdominal radiograph were excluded. Children were divided into control and intussusception groups according to whether the US results confirmed the presence of ileocolic intussusception (Fig. 4). In the intussusception group in the presence of recurrent intussusception, the radiograph scan from the time of initial diagnosis was used. Because of the asymmetrically larger number of patients in the control group compared with the intussusception group seen in real practice, we placed all of the children who had intussusception during the study period in the intussusception group and consecutive children without intussusception in twice number of intussusception group from December 2017 for the algorithm to recognize the difference in morphology with and without intussusception.

Initial supine abdominal radiographs performed prior to US were saved in DICOM format using the Picture Archiving and Communication System (PACS). Using Medical Image Processing, Analysis, and Visualization software (version 8.0.2; MIPAV; Center for Information Technology, National Institutes of Health, Bethesda, MD, USA), one experienced pediatric radiologist draw rectangular regions of interest (ROI) on the images of patients’ right abdomens, from the right diaphragm to the right iliac crest levels, lateral to the vertebral bodies (Fig. 1a). This was performed so that the algorithm would first find the anatomical region where intussusception was commonly observed and then focus on characterization of the detected region to determine the presence of intussusception. Annotations of the presence or absence of intussusception were given for each radiograph.

### Preprocessing of medical images

We applied brightness normalization to promote the rapid learning of the deep learning algorithm, because a radiograph does not have offer absolute units of brightness such as the Hounsfield units seen in computed tomography. First, using z-score normalization, the numbers of each pixel of the radiograph stored in a DICOM file were converted into z-scores, as follows:

$${\rm{z}}=\frac{x-m}{\delta }$$

where z is the z-score value, x is the pixel value, m is the mean pixel value, and δ is the standard deviation.

**Figure 5:**
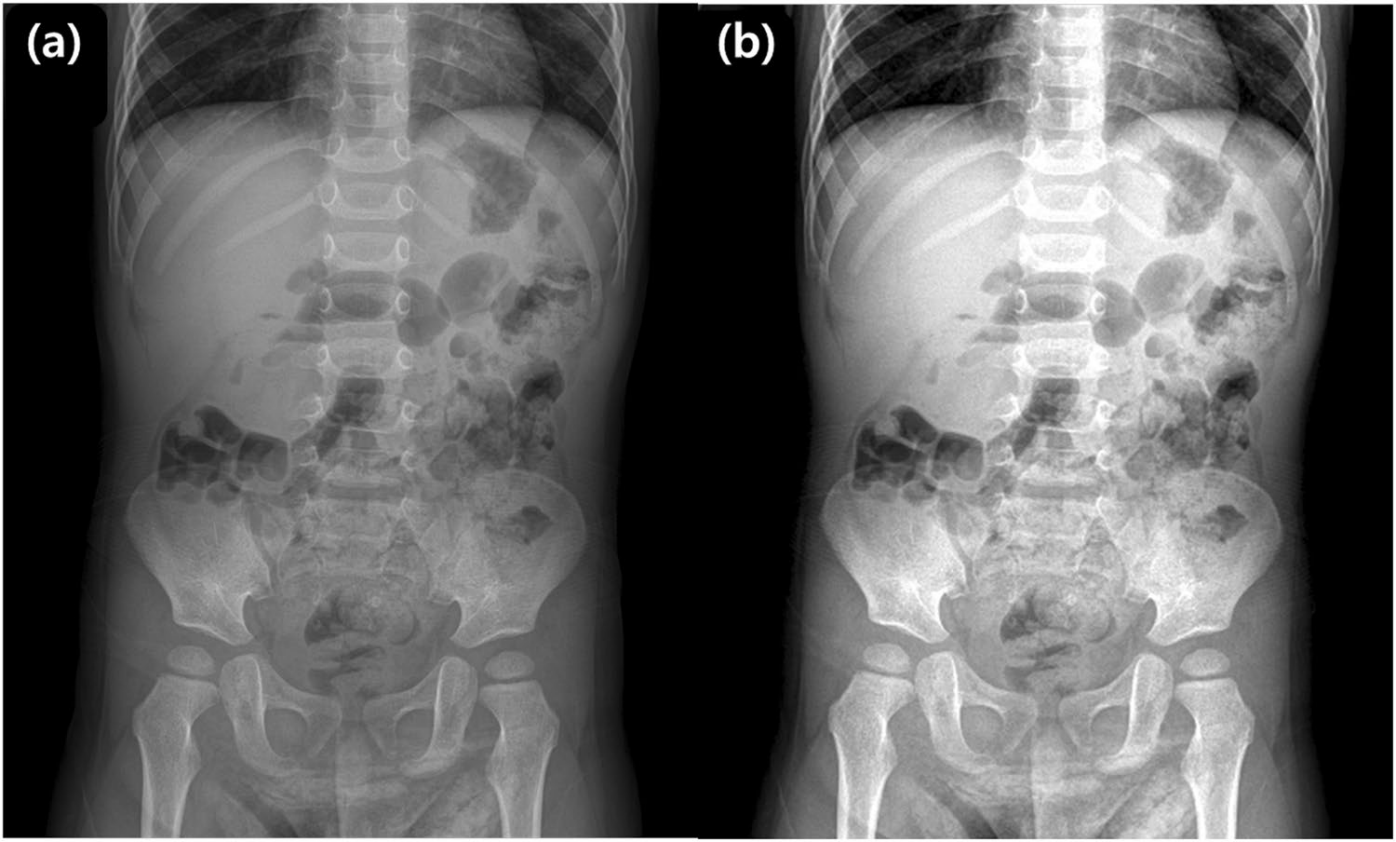
Image preprocessing steps. Image preprocessing steps were performed to promote the rapid learning of deep learning by increasing uniformity between images. (**a**) Raw DICOM image and z-score normalized image. Since the original DICOM image had 16-bit brightness levels and did not have a reference point, the original image was converted to a normalized image by z-score normalization. The raw DICOM image and z-score normalized image were the same image with only the brightness range changed. (**b**) An interval of (−1 to 1.5) was selected for the z-score values and areas outside the interval were clipped to the interval edges. We rescaled the z-score values from (−1 to 1.5) to the range of (0 to 255) to save this image in standard image format (PNG).

Given an interval of (−1 to 1.5), z-score values outside the interval were clipped to the interval edges. We then linearly scaled the z-score values between (−1 to 1.5) to between 0 and 255 and stored them in PNG image format for training. The used range (−1, 1.5) was set by the two pediatric radiologists in consensus, with the widest range being sufficient enough to observe the soft tissue and bowel pattern of the abdomen and the smallest brightness step being sufficient enough to distinguish anatomical structures (Fig. 5). Additionally, the same preprocess was applied to the images used in the validation phase for deep learning prediction.

### Training process of the deep learning algorithm

The You-Only-Look-Once (YOLO)v3, which is a state-of-the-art deep learning model, was used for region detection and classification using the implementation proposed by its original inventor (https://github.com/pjreddie). This model has shown fast detection in previous studies^21–25^. The prepared images were used to develop a YOLOv3-based deep learning algorithm to automatically recognize the rectangular area of the right abdomen as a ROI and to diagnose intussusception in the rectangular area. The YOLOv3-based deep learning algorithm was implemented in PowerEdge T630 (Dell, Round Rock, TX, USA) with four Titan-V graphic cards (NVIDIA, Santa Clara, CA, USA) hardware and Linux Ubuntu 16.04.4 LTS, NVIDIA driver version 396.26, CUDA version 9.1 software. Since the original implementation of YOLOv3 is written in C++ programming language, we executed our deep learning model using the same language. In addition, programs related to preprocessing and postprocessing were written using Python 3.6 (https://www.python.org) and Anaconda (https://www.anaconda.com).

The training set consisted of images from 242 patients with intussusception and images from 439 patients without intussusception. The number of images in each class was about twofold different, and there was a possibility of learning deterioration due to class imbalance. Therefore, the images of the intussusception class were augmented and increased by a factor of two before training, resulting in a total of 923 images [i.e., intussusception (n = 484) and non-intussusception [(n = 439)]. After augmentation, 93 images (10% of 923 images) were used as the tuning set, and 830 images were used for training. We used the transfer learning technique, incorporating convolutional weights that were pretrained on ImageNet to compensate for the relatively small number of training materials.

During training, real-time augmentation for each input image was performed by the model to continuously change the brightness, color, and size of the original image within a specified range in real time to increase the diversity of training materials and obtain better generalizability. The augmentation parameters included saturation, exposure, hue, scales, and jitters according to the default settings of the YOLOv3 model. Saturation and exposure were randomly selected in the range of (−50%, 50%). Hue and scales were randomly selected in the range of (−10%, 10%). Jitter was randomly selected within 30%. The deep learning model was trained using 50,000 iterations, which corresponds to approximately 14,000 epochs. The trained model at the 5,600^th^ iteration (1,727 epochs) showed the highest mean average accuracy. Thus, this trained model was selected as the final one to be used. Details on the training parameters and learning curves are presented in Supplementary Materials 1 and 2.

### Validation

For internal validation, we performed a 5-fold cross validation. The training set was randomly divided into five subsets so that the ratio of intussusception cases and control cases in each subset remained the same as the original training set. Additionally, the average sensitivity and specificity of the 5-fold cross validation results using the training set were presented and compared with the test set results.

For the temporally independent validation set, children (≤5 years old) who underwent abdominal US due to suspicion of intussusception following supine abdominal radiographs according to clinical demands from January to August 2018 were included (Fig. 4). In the same manner, we placed all children who had ileocolic intussusception during the study period into the intussusception group, while twice that number of children from among those who did not have intussusception from January 2018 was included in the control group.

We evaluated diagnostic performances of the algorithm and compared with those of four radiologists with different levels of experience. The DICOM files of the validation set were converted to PNG-format files through the same preprocessing process as used during algorithm training. The algorithm predicted the presence of intussusception by inputting images one-by-one. The model presented confidence scores by multiplying the probability of an object’s presence in the ROI and the class probability. In this study, the class was defined in a binary manner and the class with the higher probability among the two classes (intussusception or not) was reported as the final class with its probability. Details and an example of the YOLOv3 prediction output are presented in Supplementary Materials 3.

The four radiologists were comprised of two residents with two and four years of experience in radiology, respectively and two board-certified pediatric faculty members with seven and nine years of experience in radiology, respectively. We included radiologists with different levels of experience because we wanted to know whether there was a real difference in the detection rate of intussusception using radiographs and whether performances significantly differed between radiologists and the algorithm according to experience level, as was seen in other studies^11,26^. The abdominal radiographs were saved in JPG format by another radiologist and presented in random order to the four radiologists. The four radiologists then independently annotated the presence or absence of intussusception in each radiograph while blinded to the subjects’ clinical information.

### Statistical analysis

Statistical analyses were performed using SAS Analytics version 9.4 (SAS Institute, Cary, NC, USA). The sensitivity and specificity of the algorithm and radiologists were evaluated, and diagnostic performances were compared using logistic regression with a generalized estimating equation. A p-value < 0.05 was considered to indicate statistical significance.

## Supplementary information

Supplementary materials

## Data Availability

The datasets generated during and/or analyzed during the current study are available from the corresponding author on reasonable request.
