# Supplementary material for: Performance of deep learning-based algorithm for detection of ileocolic intussusception on abdominal radiographs of young children
Source: Sci Rep. 2019 Dec 19;9:19420. doi: 10.1038/s41598-019-55536-6 (PMC6923478; doi:10.1038/s41598-019-55536-6)
Supplement: Supplementary file 1 — Supplementary materials [file 41598_2019_55536_MOESM1_ESM.pdf]

Performance of deep learning-based algorithm for detection of ileocolic intussusception on abdominal radiographs of young children

Sungwon Kim, Haesung Yoon, Mi-Jung Lee, Myung-Joon Kim, Kyunghwa Han, Ja Kyung Yoon, Hyung Cheol Kim, Jaeseung Shin, Hyun Joo Shin*

Department of Radiology, Severance Hospital, Research Institute of Radiological Science, Center for Clinical Imaging Data Science, Yonsei University College of Medicine,

50-1 Yonsei-Ro, Seodaemun-Gu, Seoul 03722, Korea

Supplementary Materials

1. Training parameters

The parameters for training the YOLOv3 model were as follows:

Images inputted during the training process were randomly converted according to augmentation parameters. The augmentation parameters included saturation, exposure, hue, scales, and jitter. Saturation and exposure were randomly selected in the range of (-50%, 50%). Hue and scales were randomly selected in the range of (-10%, 10%). Jitter was randomly selected within 30%.

```
[net]
# Training
batch=64
subdivisions=16
width=416
height=416
channels=3
momentum=0.9
decay=0.0005
angle=0
saturation = 1.5
exposure = 1.5
hue=.1

learning_rate=0.00025 # for 4 GPUs
burn_in=4000
max_batches = 50200
policy=steps
steps=40000,45000
scales=.1,.1

[yolo]
jitter=.3
random=1
```

2. Learning curves

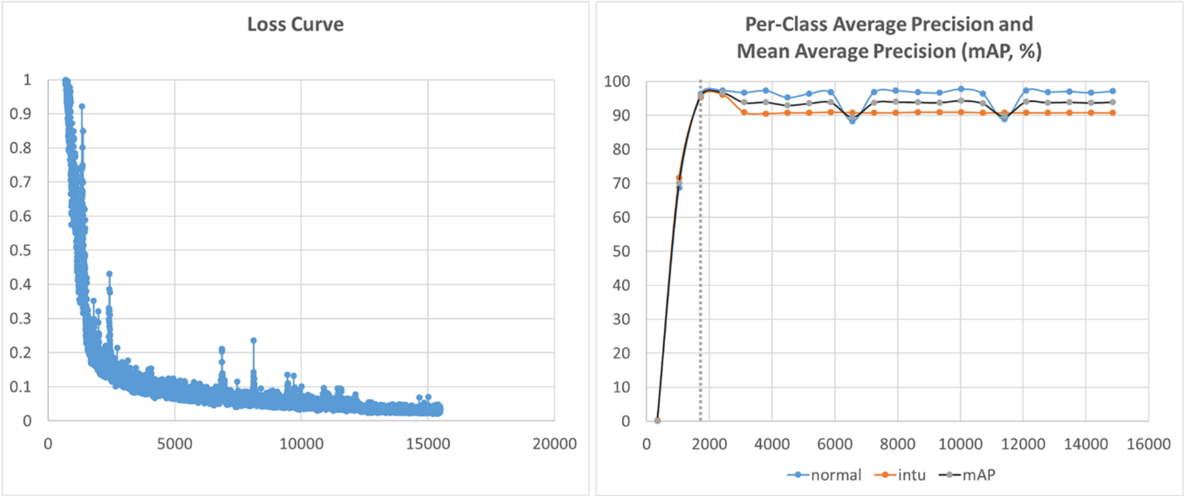

3. Example of YOLOv3 prediction output

YOLOv3 supports both image and text formats for prediction outputs, but supports only the text-form output for batch prediction. The text-form output provides the location (ROI) and class probability of the object found, as follows. If two classes were predicted with each class probability, the class with the higher class probability was considered the final decision for the radiograph. We converted this text information into an image that the radiologist could easily comprehend. The generated image and the text-form output of YOLOv3 prediction contained the exact same information.

```
Total BFLOPS 65.297
 seen 64
Enter Image Path: /home/####/image/000037.png: Predicted in 0.000000 milli-seconds.
1intu: 53%     (left_x:   49   top_y:   49   width:   127   height:   222)
```

0normal: 68% (left_x: 55 top_y: 29 width: 127 height: 250)

Enter Image Path: /home/####/image/000038.png: Predicted in 0.000000 milli-seconds.

0normal: 97% (left_x: 67 top_y: 10 width: 119 height: 286)

Enter Image Path: /home/####/image/000039.png: Predicted in 0.000000 milli-seconds.

0normal: 100% (left_x: 41 top_y: 35 width: 153 height: 256)

Enter Image Path: /home/####/image/000040.png: Predicted in 0.000000 milli-seconds.

1intu: 34% (left_x: 28 top_y: 20 width: 166 height: 264)

0normal: 95% (left_x: 30 top_y: 17 width: 150 height: 255)

Enter Image Path: /home/####/image/000041.png: Predicted in 0.000000 milli-seconds.

0normal: 99% (left_x: 28 top_y: 18 width: 148 height: 251)
